# Laparoscopic Findings Revealing a Mature Ovarian Teratoma Initially Diagnosed as a Mesenteric Tumor: A Case Report

**DOI:** 10.7759/cureus.116017

**Published:** 2026-09-09

**Authors:** Edgar Alexis Flores García, Emiliano De La Torre Pacheco, Abigail Diaz Alba, Francisco J Rivera Vazquez, Hector X Rodriguez, Jenniffer Luján Hurtado, Oscar E Coronado Padilla

**Affiliations:** 1 Surgery, Hospital General Nuevo Gómez Palacio, Gómez Palacio, MEX

**Keywords:** acute abdominal pain, dermoid cyst, laparoscopic teratoma, mature ovarian teratoma, mesenteric mass

## Abstract

Mature ovarian teratomas are the most common benign germ cell tumors of the ovary and usually affect women of reproductive age. Their clinical presentation is variable, and in some cases, imaging findings may be misleading, complicating the preoperative diagnosis. We report a case of an 18-year-old female patient who presented to the emergency department with acute abdominal pain. Imaging studies revealed a large intra-abdominal mass initially interpreted as originating from the mesentery. Due to persistent symptoms and diagnostic uncertainty, surgical exploration was performed. Intraoperatively, the mass was identified as an ovarian lesion, and complete resection was achieved. Histopathological examination unexpectedly confirmed the diagnosis of a mature cystic teratoma without malignant features. The postoperative course was favorable, and the patient recovered without complications. This case highlights the diagnostic challenges associated with atypical presentations of ovarian teratomas and underscores the importance of considering gynecologic pathology in the differential diagnosis of abdominal masses in young women, even when imaging suggests an extragonadal origin. Surgical exploration remains essential when clinical presentation involves severe symptoms or diagnostic ambiguity, allowing both definitive histopathological diagnosis and resolution of symptoms.

## Introduction

Mature ovarian teratomas, also known as dermoid cysts, are the most common germ cell tumors of the ovary and account for approximately 10-20% of all ovarian neoplasms [1]. They predominantly occur in women of reproductive age and are composed of well-differentiated tissues derived from at least two of the three germ cell layers, frequently containing adipose tissue, hair, and calcifications [2].

Imaging plays a fundamental role in the preoperative evaluation of adnexal and intra-abdominal masses. Computed tomography (CT) is particularly useful for identifying macroscopic fat and calcified components, which are considered characteristic imaging features of mature cystic teratomas [3]. However, atypical presentations, unusual anatomic relationships, or large tumor size may lead to diagnostic uncertainty and misinterpretation, occasionally resulting in an incorrect preoperative diagnosis [4].

Accurate identification of mature ovarian teratomas is clinically important as these lesions are benign and associated with an excellent prognosis following complete surgical excision [5]. An incorrect preoperative diagnosis may influence surgical planning and delay appropriate management.

In this report, we present a case of a mature ovarian teratoma in an 18-year-old patient that was initially interpreted on CT as a mesenteric mass, highlighting the diagnostic pitfalls of cross-sectional imaging and emphasizing the importance of radiologic-surgical correlation.

## Case presentation

An 18-year-old female patient with no relevant past medical history was referred from the emergency department due to severe abdominal pain localized to the left iliac fossa and pelvic region. Physical examination revealed localized tenderness in the left lower quadrant without signs of peritoneal irritation. Laboratory studies, including complete blood count and inflammatory markers, were within normal limits. The only abnormal finding throughout the clinical evaluation was persistent pelvic and left iliac fossa pain.

Given the severity of the acute abdominal pain, the need for rapid diagnostic evaluation in the emergency setting, and to avoid delays while awaiting renal function panel results for IV contrast, a non-contrast abdominal CT scan was performed as the initial imaging modality. Transpelvic/transvaginal ultrasonography was not performed prior to surgery due to the clinical urgency and the initial interpretation of the lesion as an extrapelvic mass. The scan demonstrated a well-defined heterogeneous mass in the lower abdomen that appeared anatomically distinct from the adnexa and was surrounded by mesenteric structures, leading to its initial interpretation as being of mesenteric origin (Figure 1). Notably, the presence of internal fat attenuation and focal calcifications ruled out a simple mesenteric cyst and raised a differential diagnosis that included a mesenteric teratoma, liposarcoma, gastrointestinal stromal tumor (GIST) with regressive changes, or an exophytic/pedunculated ovarian mature cystic teratoma.

**Figure 1:**
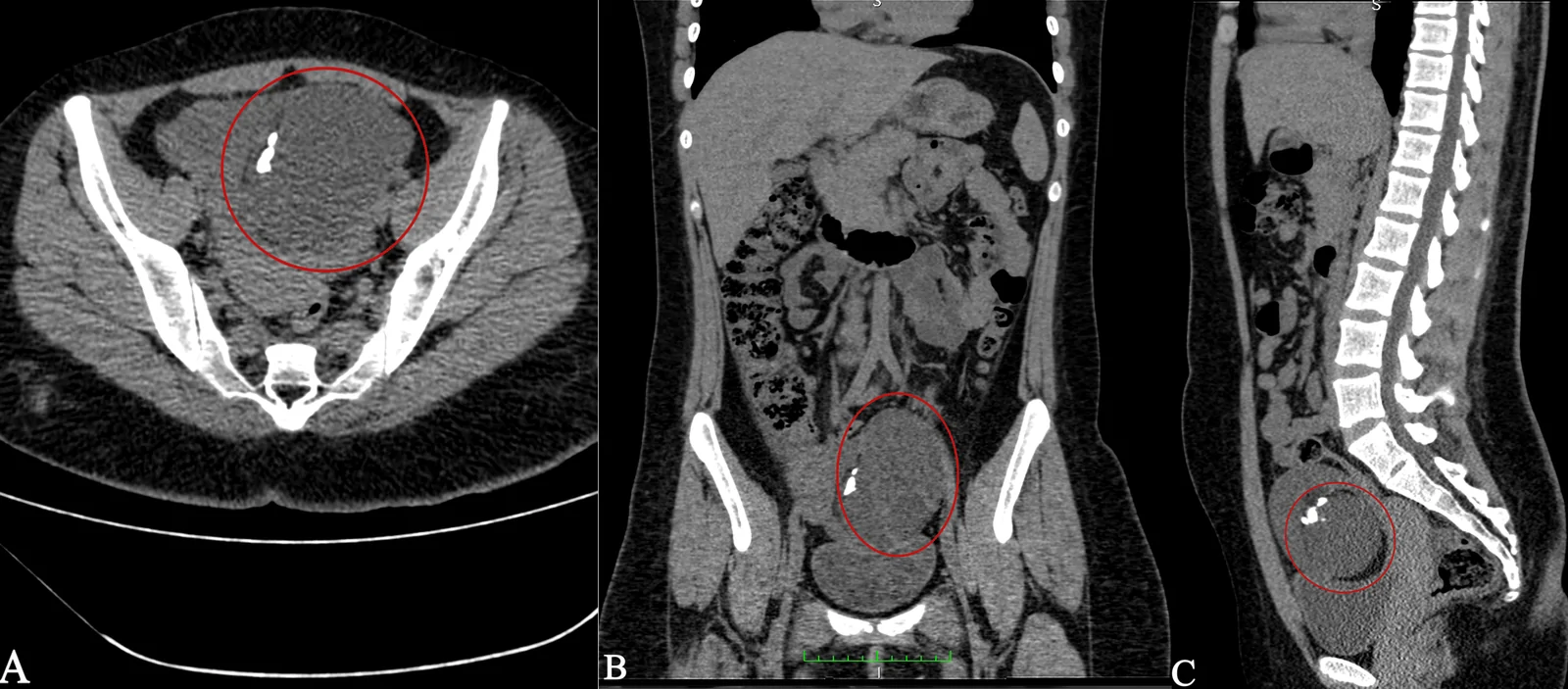
Computed tomography (CT) images demonstrating an intra-abdominal mass. (A) Axial view, (B) coronal view, and (C) sagittal view showing a well-defined heterogeneous mass (red circle) containing internal calcifications.

Due to the diagnostic uncertainty and the patient’s ongoing symptoms, a laparoscopic surgical approach was undertaken. Intraoperatively, the mass was identified as originating from the ovary rather than the mesentery. To prevent intraoperative spillage of cystic contents into the peritoneal cavity and minimize the risk of chemical peritonitis, the resected mass was immediately placed into an impermeable laparoscopic retrieval bag (endobag). To facilitate specimen removal, the lesion was incised intraoperatively inside the bag (Figure 2). Controlled decompression and piecemeal extraction of the tumor contents - including hair and calcified components - were performed exclusively within the retrieval bag through a modified port site (Figures 3-4). Following complete removal of the specimen, extensive warm saline peritoneal lavage was performed to ensure no residual material remained in the abdominal cavity. Macroscopic examination revealed abundant hair of varying coloration and calcified components, findings consistent with a mature ovarian teratoma (Figure 5). Subsequent histopathological examination confirmed the diagnosis of a mature cystic ovarian teratoma without evidence of malignancy.

**Figure 2:**
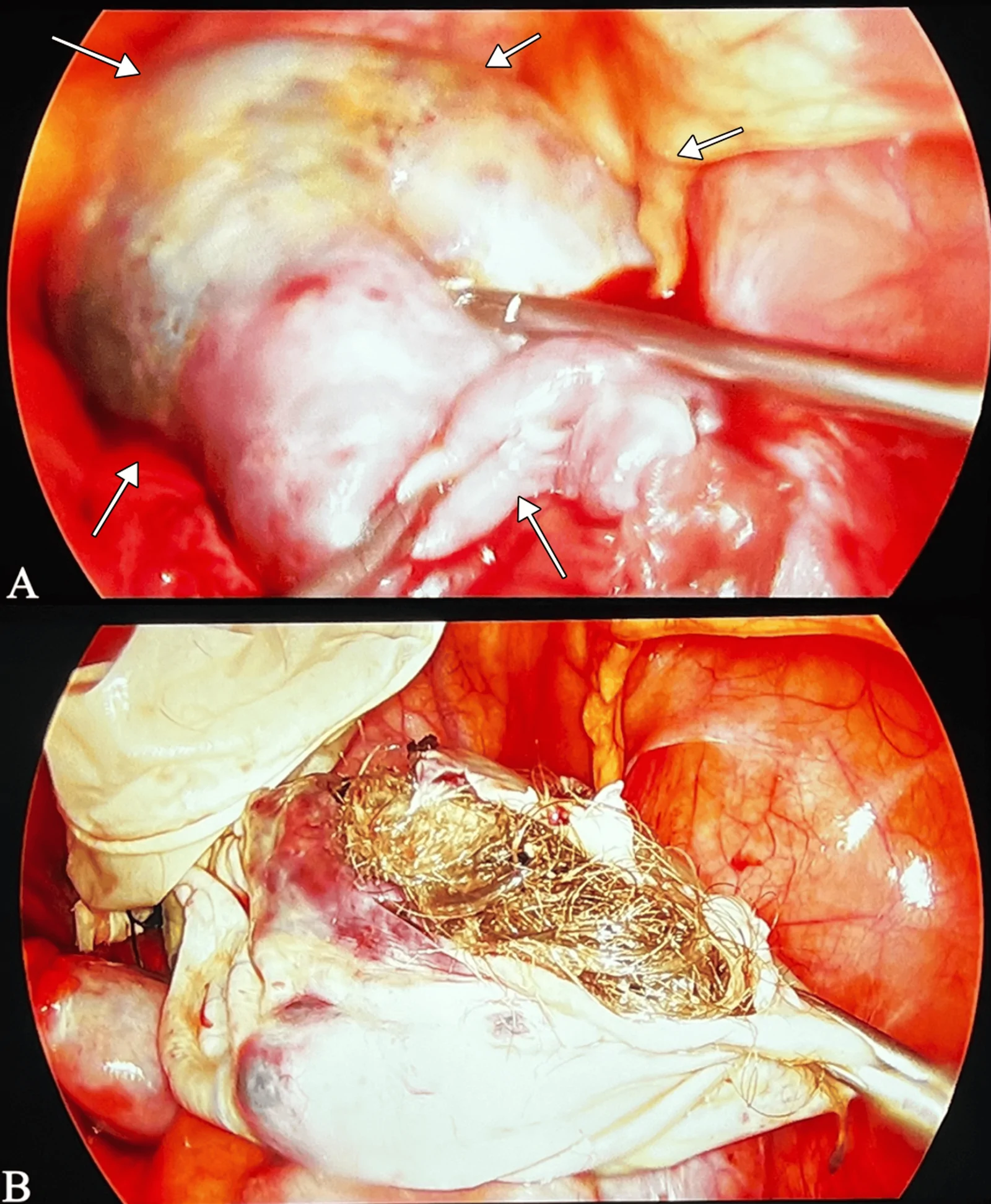
Intraoperative laparoscopic views of the ovarian mass. (A) Enlarged ovary after surgical dissection, with the mass highlighted by white arrows. (B) Opened ovarian mass revealing internal contents, predominantly hair, consistent with a mature cystic teratoma.

**Figure 3:**
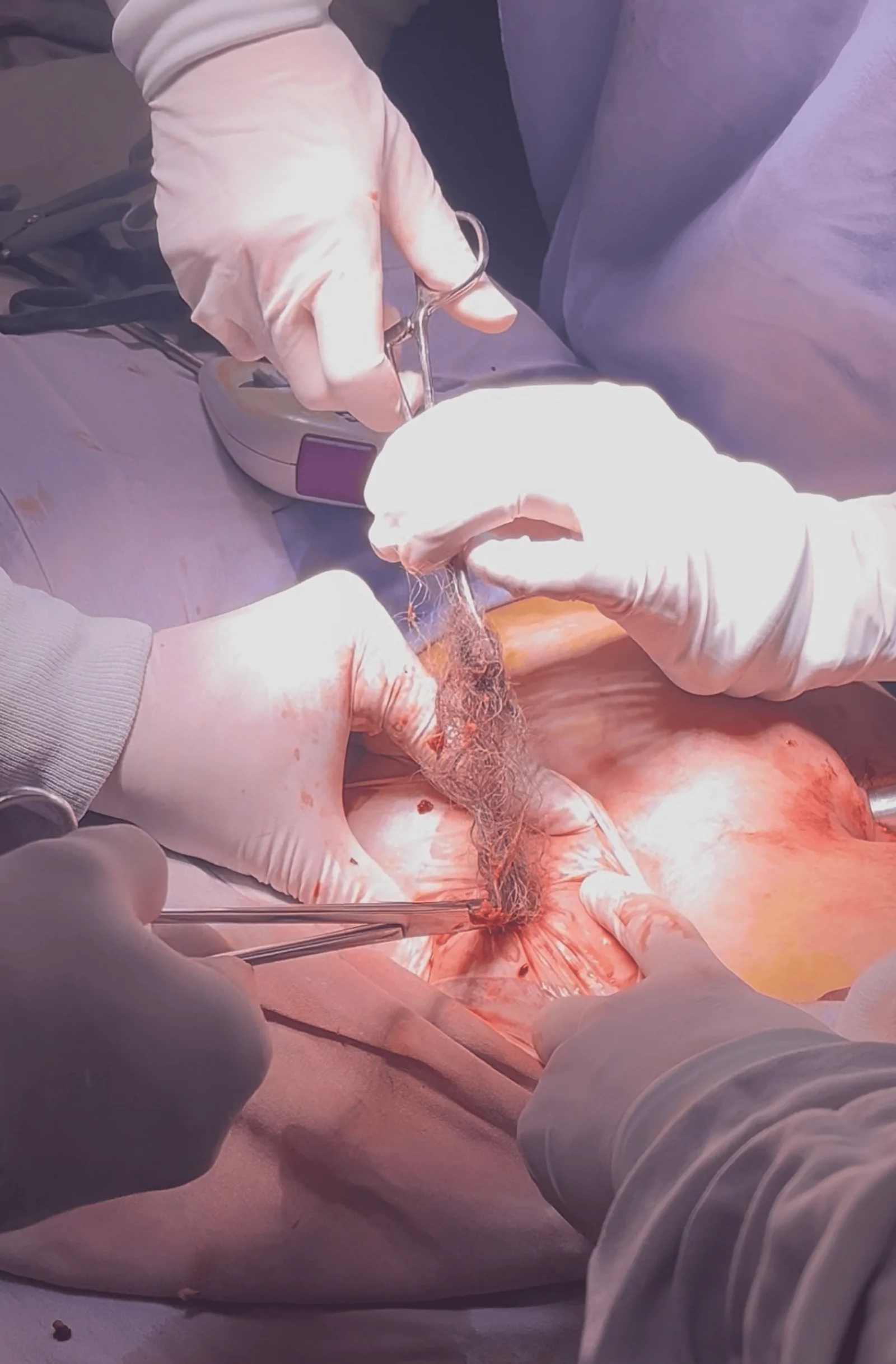
Intraoperative laparoscopic image showing extraction of hair from the ovarian teratoma through one of the laparoscopic ports (Covidien (Medtronic), Mansfield, USA).

**Figure 4:**
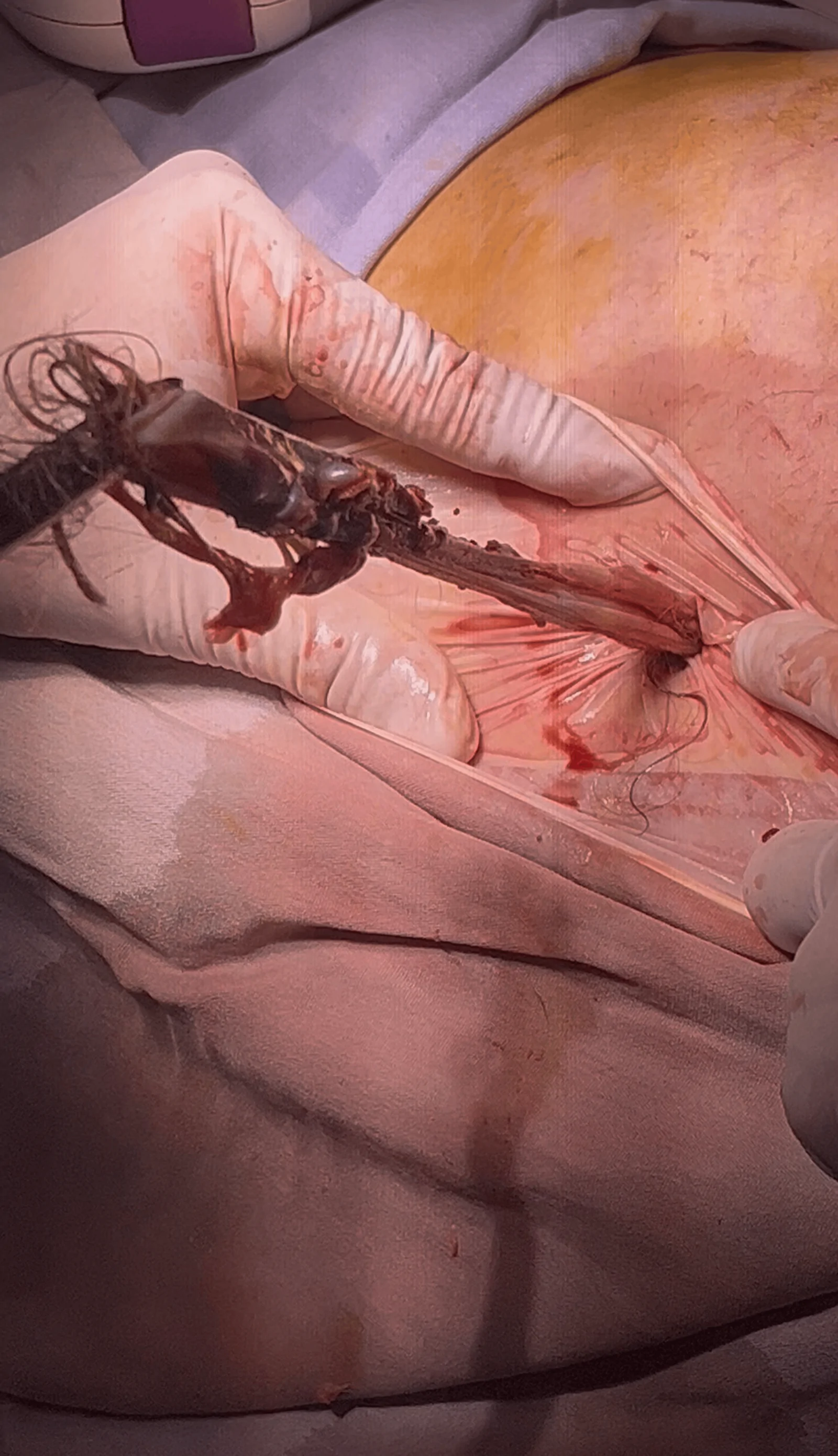
Intraoperative laparoscopic image showing piecemeal extraction of tumor tissue from the ovarian teratoma through a laparoscopic port (Covidien (Medtronic), Mansfield, USA).

**Figure 5:**
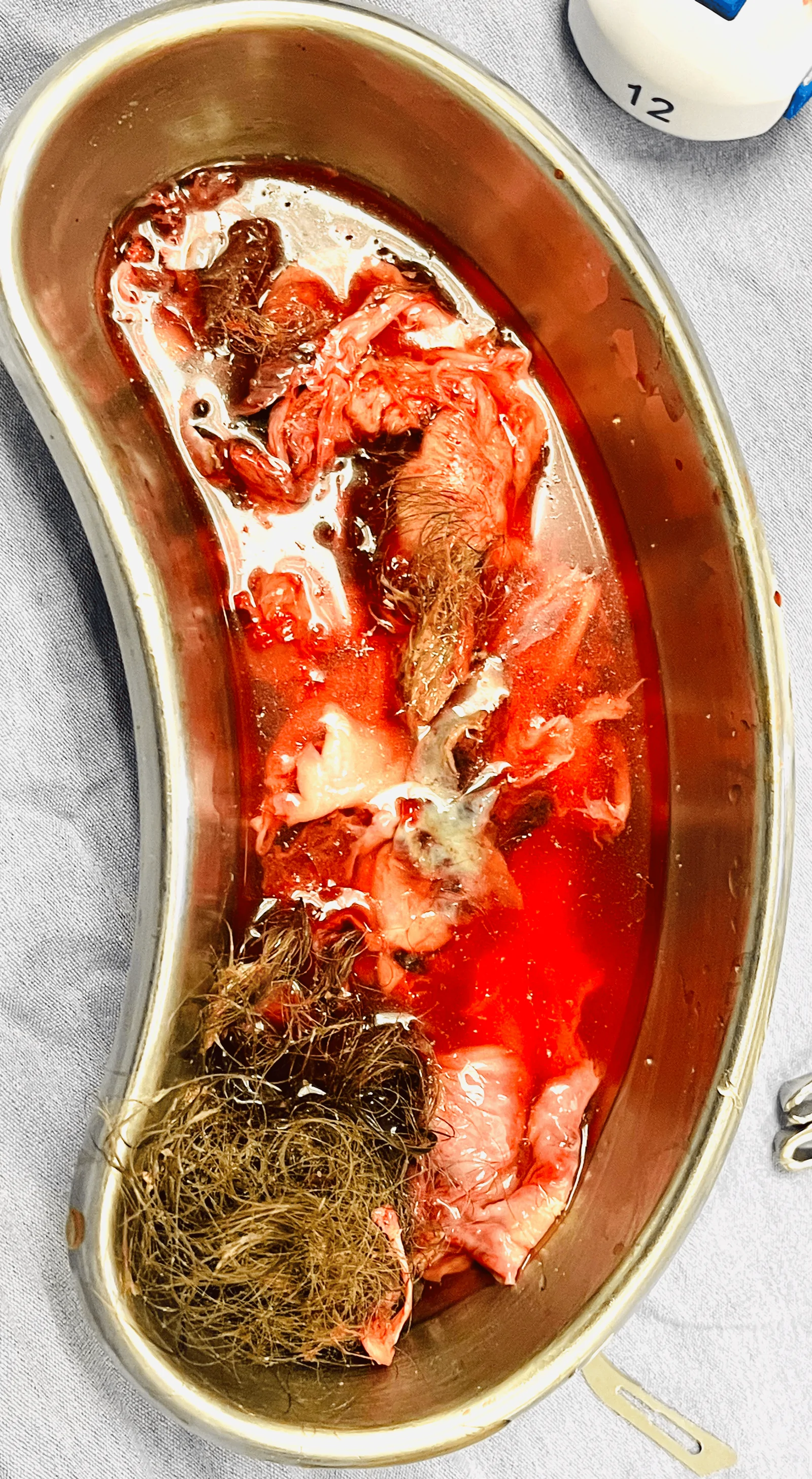
Gross examination of the surgical specimen within a sterile basin. The image displays a mature cystic teratoma post-excision, featuring prominent ectodermal components, including terminal hair follicles and areas of calcification suggestive of osseous or dental tissue.

The patient had an uneventful postoperative course and was discharged in stable condition.

## Discussion

Mature ovarian teratomas are benign germ cell tumors characterized by the presence of well-differentiated tissues originating from multiple germ layers. Large clinicopathologic series have demonstrated that these lesions are among the most frequent ovarian neoplasms and are commonly diagnosed in young women, often during the second and third decades of life [6]. Despite their benign nature, their clinical presentation may vary widely, ranging from incidental findings to acute abdominal pain due to torsion, rupture, or mass effect.

In young female patients presenting with a heterogeneous lower abdominal mass containing fat and calcifications, the differential diagnosis should not be limited to adnexal lesions. Although mature cystic teratomas are the most frequent etiology, primary retroperitoneal or mesenteric teratomas, pelvic liposarcomas, mesenteric panniculitis, and calcified GISTs must also be considered. Distinguishing between a primary mesenteric lesion and an exophytic or torsion-prone ovarian mass can be particularly challenging when the ipsilateral ovary is compressed or poorly delineated on cross-sectional imaging.

From an imaging perspective, CT and magnetic resonance imaging (MRI) are highly reliable for identifying classical features of mature ovarian teratomas, such as macroscopic fat attenuation, Rokitansky nodules, tooth-like calcifications, and fat-fluid levels [7]. However, atypical radiological presentations can significantly hinder accurate preoperative diagnosis. These atypical features include predominantly cystic or solid appearances with minimal or absent macroscopic fat, extensive rim or coarse calcifications masking internal architecture, palm-tree or floating hair signs, and palm-leaf-like septations [8]. Furthermore, when teratomas undergo severe ischemia or necrosis due to chronic torsion, or when they feature prominent exophytic growth with a thin vascular pedicle, the normal ipsilateral ovary may not be distinctly visualized. In such scenarios, the lesion often appears anatomically disconnected from the adnexa, falsely mimicking primary mesenteric, retroperitoneal, or gastrointestinal neoplasms [8,9].

Radiologic differentiation between adnexal and mesenteric tumors may be especially challenging when normal ovarian anatomy is not clearly visualized. Awareness of these potential pitfalls is essential to avoid misinterpretation and to guide appropriate surgical planning [9]. In such cases, surgical exploration remains both a diagnostic and therapeutic tool.

Laparoscopic management is currently considered the preferred approach for mature ovarian teratomas in appropriately selected patients, offering the advantages of reduced postoperative pain, shorter hospital stay, and faster recovery [10]. Careful intraoperative handling is required to prevent spillage of cyst contents, which may lead to chemical peritonitis. Established management guidelines emphasize complete excision and thorough peritoneal lavage when intraoperative rupture occurs [11].

Although malignant transformation is rare, occurring in a small percentage of cases, histopathological examination is mandatory to confirm the benign nature of the lesion and exclude malignancy [12]. In the present case, laparoscopic exploration allowed definitive diagnosis and successful treatment, underscoring the importance of correlating radiologic findings with intraoperative observations in cases of diagnostic uncertainty.

Finally, this case highlights critical educational points regarding the diagnostic approach to pelvic and abdominal masses in young women. Pelvic ultrasonography - particularly transvaginal or transabdominal ultrasound - remains the first-line, non-invasive modality of choice for assessing adnexal lesions, capable of detecting characteristic acoustic shadowing and hair-fluid interfaces. Furthermore, contrast-enhanced CT or MRI provides superior soft-tissue resolution to delineate pedicle vascularity and trace anatomical origins. The reliance solely on non-contrast CT in the emergency setting represented a diagnostic limitation in this case. Incorporating pelvic ultrasound and contrast-enhanced imaging into the initial workup of lower abdominal masses in young female patients is highly recommended to avoid misdiagnosis and optimize preoperative surgical planning.

## Conclusions

Mature ovarian teratomas are common benign ovarian tumors with characteristic imaging features; however, atypical presentations may lead to diagnostic challenges. This case illustrates how a mature ovarian teratoma can mimic a mesenteric mass on CT, emphasizing the limitations of preoperative imaging in certain clinical scenarios. Laparoscopic exploration played a crucial role in establishing the correct diagnosis and achieving definitive treatment. Awareness of this potential diagnostic pitfall is essential to ensure appropriate surgical planning and optimal patient outcomes, particularly in young patients presenting with acute abdominal pain.
